# 

**Published:** 1969-07

**Authors:** 


### BRISTOL MEDICO-CHIRURGICAL JOURNAL

*(Incorporating the Medical Journal of the South-West)*

Established 1883

Vol. 84 (iii) July 1969 No. 311

### *Contents*

Page

ANAPHYLACTOID PURPURA, M. E. R. Stoneman ... ... ... 61

MYELOID LEUKAEMOID REACTION IN A SURGICAL CASE, M. M. Ashraf ... ... ... ... ... ... ... ... 72

SIXTY YEARS OF STOKE PARK HOSPITAL, J. Jancar ... ... 77

SOUTH WEST ORTHOPAEDIC CLUB ... ... ... ... ... ... 97

OBITUARY, J. A. POCOCK ... ... ... ... ... ... 100

RECENTLY APPOINTED CONSULTANTS ... ... ... ... 103

***PUBLISHED QUARTERLY***

***ANNUAL SUBSCRIPTION 16/- POST FREE***

### BRISTOL MEDICO-CHIRURGICAL SOCIETY

President 1968/1969 : Mr. A. L. Eyre-Brook

President elect : Dr. F. J. W. Lewis

Honorary Secretary : Dr. I. S. Bailey, 7 Percival Road, Bristol 8.

Honorary Treasurer : Dr. J. Macrae, Ham Green Hospital, Pill.

The Society was founded in 1874. In 1883 publication of this Journal began, and has continued quarterly since then. During the years 1949 to 1962 it appeared under the title of "The Medical Journal of the South West".

The Society founded a medical library in 1890, which in 1892 was combined with that of the Bristol Medical School. This became the University of Bristol Medical Library in 1925, and an agreement in that year confirmed the privilege of Members to use the University Medical Library.

### THE BRISTOL MEDICO-CHIRURGICAL JOURNAL

*Editorial Committee*

*Editors*

Dr. N. J. Brown, Southmead Hospital, Bristol.

Dr. A. B. Raper, Royal Infirmary, Bristol

*Members*

Mr. H. B. Griffith

Dr. M .B. Lennard

Dr. A. E. Read

Mr. J. B. M. Roberts

Professor R. C. Wofinden

Dr. D. W. Wright

*Secretary*

Mr. A. E. S. Roberts, The Library, Medical School, University Walk, Bristol, 8.

The Hon. Treasurer and Hon. Secretary of the Society.

### NOTICE TO CONTRIBUTORS

The Journal is especially concerned to provide a place for the recording of medical thought, interests, and practice in and around Bristol. It therefore welcomes articles that, as well as being of immediate interest to members, will document the local and contemporary medical scene for our successors.

Original articles are invited provided that they have not been published elsewhere. References in the text should be by author's name, with year of publication in parenthesis; they should be listed at the end in alphabetical order. Illustrations should be supplied ready for reproduction. Line drawings should be in indian ink on firm white paper or board. The author will be informed if it is found necessary to ask him to pay for the making of blocks.

The following contributions will be especially welcome: notes of forthcoming events, meetings held, degrees conferred, staff appointments, honours and public appointments and other local news.

### NOTICE

### The late Mr. John Pocock, F.R.C.S.

Friends of the late Mr. John Pocock, F.R.C.S., who was a Consultant Surgeon at Southmead Hospital, Bristol, for many years, wish to remember him in a practical fashion which will benefit the medical profession and the general public.

Those who knew him believe that a contribution to medical education would be a memorial he would have liked best.

Provision for a new Medical Library is being made in the new Medical Teaching Unit at Southmead Hospital and it is intended to form a Libraries Fund, named after Mr. Pocock, from which income will be available for the purchase of suitable books.

Many readers will have known Mr. Pocock and will wish to be associated with his memorial.

Contributions should be sent to the Honorary Treasurer, The John Pocock Memorial Fund, c/o Southmead Hospital, Bristol BS10 5NB.

Printed by

F. Bailey & Son, Dursley, Glos.

